# Going for brokerage: strategies and strains in commercial healthcare facilitation

**DOI:** 10.1186/s12992-020-00578-z

**Published:** 2020-05-29

**Authors:** Benjamin M. Hunter

**Affiliations:** 1 Department of International Development, King’s College London, London, UK; 2 Department of International Development, University of Sussex, Brighton, UK

**Keywords:** India, UK, Healthcare, Facilitation, Medical tourism, Concierge, Commercialisation, Markets, Brokerage

## Abstract

**Background:**

The formation of domestic and global marketplaces during the past 50 years has opened up new commercial opportunities for third-party activity in healthcare systems. Commercial mediation of access to healthcare is one recent area of activity that sees companies and individuals offering to organise healthcare and travel in return for payment. With varying degrees of control over the location, type, cost and experiences of healthcare provisioning, these intermediaries occupy potentially influential positions in healthcare systems and yet much of their work is poorly understood.

**Methods:**

Drawing on social science theories of brokerage, this article presents a novel analysis of commercial healthcare facilitation. It focuses on facilitation companies and their workers as central, intermediating actors for people to access healthcare in markets characterised by complexity. Semi-structured interviews were conducted with people working in domestic and international healthcare facilitation in London and Delhi, and data were analysed using a framework approach that emphasises the structural features and personal agencies for this area of work.

**Results:**

Findings point to an institutional environment for commercial healthcare facilitation marked by competition and the threat of obsolescence. The activities of rivals, and the risk that users and providers will bypass intermediaries, compels facilitation companies to respond strategically and to continuously pursue new populations and activities to mediate – to go for broke. These pressures percolate into the lives of people who perform facilitation work and who describe a physical and mental burden of labour incurred by onerous processes for generating and completing facilitation work. The need for language interpretation services introduces an additional set of relations and has created further points of tension. It is an environment that engenders mistrust and anxiety, and which incentivises exploitation and a commodification of users whose associated commissions are highly prized.

**Conclusion:**

Brokerage analysis provides valuable insights into the strategies and strains for commercial mediation of access to healthcare, and the findings indicate opportunities for further research on the contributions of interpreters, diplomatic and business networks, and new technologies, and on the growth of new forms of mediation in domestic and overseas settings.

## Background

Restructuring within healthcare systems in recent decades has seen the expansion of market-based provisioning in many settings [1, 2]. It is a trend that has been accompanied by growth in cross-border travel for healthcare, as people seek care in global healthcare markets for services unavailable to them in their country of origin [3–6]. The expansion of these domestic and global markets for healthcare pose new sets of choices, and with them new bureaucratic and informational barriers and needs for accessing care [7], and this has opened up commercial opportunities for third-parties to operate as intermediaries in healthcare markets.

The emergence of commercial facilitation services has been a notable feature of healthcare in recent decades, as individuals and companies offer to organise and mediate access to healthcare in return for payments. Often the focus of these activities has been to cater to the growing number of people who cross borders to search of care: one study identified 208 web-based facilitation companies globally [8], and many more operate on an informal basis in countries such as Mexico [9], Malaysia [10] and India [11]; while some focus on global markets for specific services such as assisted reproduction [12–14] or cosmetic surgery [15]. There are also small but growing markets for facilitation services in a domestic context, for example the high-end ‘concierge’ services being offered in some countries [16].

The presence of commercial facilitation in healthcare and its potential implications for healthcare provisioning and financing warrants closer scrutiny. With some important exceptions from the study of cross-border travel for healthcare – including Snyder et al. [17], Dalstrom [9] and Hartmann [11] – researchers have tended to rely on second-hand accounts of facilitation activities using testimonies from healthcare providers [18, 19]. These analyses have adopted network-based understandings of facilitation to examine its transient social formations: the ‘stabilising and destabilising processes and connections’ ( [4], p. 133). They consider the socially structured movements of users [20] that demand continuous mobilising work [11]; and the array of actors who are drawn together to provide services, for example accommodation vendors, translators, drivers, physiotherapists and cooks who perform ancillary services [21]. There are tensions involved in these relations, as providers and intermediaries use a range of contractual and other mechanisms to associate more closely or loosely with one another for reasons of legitimacy, exclusivity and responsibility [4].

Here I use first-hand accounts, generated through interview-based research with people working in domestic and/or international healthcare facilitation in London and Delhi, in order to deepen understanding of commercial healthcare facilitation through attention to its structures, strains and the personalised effects these have. Rather than understanding and analysing healthcare facilitation in terms of its networked connections, I make the case for using a brokerage framework inspired by the social science study of markets and socio-political systems. This devotes greater consideration to the strategic decisions and personal agencies that characterise commercial healthcare facilitation. After explaining the conceptual framework for the research, I outline the methods used for data collection and analysis, and then set out a series of findings. I argue that facilitation companies operate in competitive markets, characterised by alternative and rival channels for accessing healthcare, and that companies are compelled into a continuous pursuit of expansion - to go for broke. Closer consideration of the work of facilitation reveals ways in which the pressures of competition and expansion percolate into the lives of people labouring in precarious arrangements and incentivise commodification of users. In the discussion I reflect on the value of the brokerage framework for studying commercial healthcare facilitation, and highlight areas for further study.

### Healthcare facilitation as brokerage

There is a large body of social science literature on brokerage which offers a basis for understanding the social relations for third party-mediated activities such as the commercial facilitation of access to healthcare. Brokerage involves the (often deliberate) positioning of third parties as intermediaries in exchange relations in ways that allow these intermediaries to create and extract value from exchange relations [22]. There is historical precedent for brokerage as a form of economic activity, from the arbitrage performed by merchants in trading networks [23], to the opportunities for mediation that arose with markets for financial products and services [24, 25]. However, such brokerage is not confined to overtly commercial exchanges and indeed much of the social science literature on brokerage considers how members of political networks leverage resources and social relations to assist certain constituencies in return for political support [26–28]. Brokerage relations repeatedly emerge in these scenarios of complexity and exclusion – where there are significant transaction costs that reduce the likelihood of direct exchanges between parties.

Transaction costs, complexity and exclusion are well-known features of healthcare’s provisioning markets [29, 30], and accordingly there are several examples of intermediation in contemporary healthcare systems. Best documented is the intermediary role for facilitation companies in cross-border travel for healthcare: these companies seek to attract potential clients through glossy websites and personal referrals [31, 32], an active social media presence [13], and partnering with medical insurance companies and large employers [33]. They offer information and arrange travel, alongside more socially attuned and customisable support like accompanying users to offer advice on how to act, travel, eat and speak [9, 19] and companionship and moral support [10]. Facilitation companies are approached by, and approach, providers with whom to partner [18], often at the large trade events that have emerged [34, 35], and seek to develop a range of possible packages and destinations to offer to prospective clients [36]. Though less well documented, a set of ‘concierge’ companies offer broadly similar mediation services but often focus within domestic (usually private) healthcare systems [16] and are an extension into healthcare of the concierge economy that caters to the consumption needs of a global elite.

The intermediary positions, and the negotiated and dynamic relations involved, mark these forms of facilitation out as systems of brokerage, and indeed the notion of the ‘broker’ has previously been used to elaborate social relations in the study of cross-border travel for healthcare. Dalstrom’s ([9], p. 25) research in Mexico employed the concept of ‘cultural brokerage’ to understand the role of intermediaries in advising travellers how to behave in an unfamiliar setting, while Snyder et al. [17] distinguished between ‘facilitator’ and ‘broker’ roles, suggesting the latter have less direct contact with healthcare users, although here I follow Skountridaki [18] and others in using ‘facilitation’ as an umbrella term for these actors. Hartmann’s [11] recent work on cross-border travel into Delhi’s healthcare system adopted an understanding of brokerage used in mobilities and migration studies which attends to the social processes for developing and maintaining relationships across borders. Here I adopt an understanding of brokerage that draws from social science literature on markets and socio-political systems and which pays close attention to strategic positioning, negotiation and personalised effects.

In earlier work, I used a novel analytical framework to study informal systems of brokerage as performed by lay community health workers in an aid-funded health voucher scheme in northern India [37]. That study showed how workers exercised varying degrees of personal agency when interpreting their roles within the context of a healthcare market; they attempted to consolidate their intermediary roles and pursue opportunities for personal benefit. In this article I build on that analysis by adapting and applying the analytical framework to the study of formalised systems of commercial healthcare facilitation in Delhi and London. Despite differing institutional contexts, these settings are similarly characterised by the existence of healthcare provisioning markets and the emergence of systems for third-party mediation.

One line of analysis that I develop further here is a concern with the labour processes in brokerage relations. While there have been calls to devote greater attention to labour in the study of health work, particularly in the context of globalisation [38], the labour performed by people working in services ancillary to healthcare has attracted less attention. Notable exceptions include research on the time-pressured sales work performed by medical representatives [39, 40] and the unhealthy working conditions for hospital cleaning staff [41]. Ancillary services like commercial healthcare facilitation play an important role in the production of healthcare and it is important to understand how structural issues affect the conditions of labour in these areas of work, and how they in turn incentivise particular behaviours that have implications for the health and wellbeing of workers and healthcare users.

## Methods

The research was conducted as part of a project on formalised systems of brokerage in healthcare, and ethics approval for the project was provided by King’s College London. Interviews were conducted during 2018 with 33 people who have detailed knowledge of commercial healthcare mediation in and around Delhi (24 respondents) and London (9 respondents), including representatives from 22 facilitation companies. These settings offer a range of specialised healthcare services and are established destinations for domestic and international healthcare users seeking care otherwise unavailable in their home locality [42]. Delhi is a global destination for relatively low-cost medical tourism [19], and attracts users from within and beyond India. London is a destination for advanced treatments, particularly for wealthy users travelling to London from countries in the Middle-East [43], but healthcare providers also offer private services to people living in the UK and there has been a trend for people living in the UK to travel to other countries to seek services that are unavailable, or considered over-priced, in the UK healthcare system.

Respondents were identified based on their geographical base of operations in and around Delhi and London, and based on their knowledge of domestic or international facilitation in the healthcare sector. The majority worked in facilitation companies: in London, seven had set up and run small facilitation companies (between one and five employees), and two worked for small companies; in Delhi, four were founders of small companies, six were founders of larger companies (greater than five employees), and several worked for larger companies - four as business development managers, one as a mid-level manager and one as a case worker. I also interviewed two people in Delhi who work in the international marketing teams of large private hospitals, two who had worked as interpreters with facilitation companies, and four people who worked in investment and consultancy industries and had knowledge of the sector.

Respondents were contacted by email or by phone, informed of the aims of the project and invited to participate by interview at a time and location of their choosing, or via Skype or phone. Before commencing each interview, the aims of the project were explained to respondents, and during the interview I took notes that summarised the discussions taking place. These notes were then written up in detail immediately after the interview. Interview questions varied depending on the professional role of the respondent, but generally related to the activities they performed, their motivations and the challenges faced personally, and broader issues faced by facilitation companies. Informal meetings with two additional respondents – one a case worker in a facilitator company and the other a community leader who regularly worked with healthcare users visiting Delhi from an Eastern African country – were written up as field-notes after those discussions took place.

Notes from the interviews were analysed using a framework approach [44]. This approach interrogates data using pre-determined questions and entails five steps: familiarisation, identification of a thematic framework, indexing, charting and interpretation. Six framework charts were created, corresponding to the six areas of interest used in an earlier analysis of brokerage relations in healthcare facilitation: activities performed by intermediaries; social relations that permit facilitation; benefits to different groups engaged in brokered relations; expansion and consolidation of intermediary positions; costs and tensions for brokerage relations, and responses to changing institutional landscapes [37]. A set of columns were added to each chart based on pre-determined categories and were added to or revised as necessary during the analysis process. I went through the interview- and field-notes and summarised any passages relevant to an analytical category - adding them to the respective column in a chart - and then used the charts to map the range of issues discussed and to find associations between charts. During analysis I identified two cross-cutting themes which are used to present the findings below: structural issues and strategic responses for facilitation companies; and the personalised strains this places on people who perform the work of facilitation.

## Results

### Structures and strategies for brokerage

#### A “saturated” market for intermediation

The core offering of commercial healthcare facilitation is to organise healthcare on behalf of others, often including a choice of providers and countries, and support with travel, accommodation, visa applications, language interpretation and tourism. Respondents reported working with people who travelled from comparatively under-resourced, or comparatively expensive, healthcare systems. The wealthiest users of their services desire high-end care and luxury travel arrangements and fund their care through personal wealth, private health insurance, or government- or employer-sponsored travel. But many respondents also catered to poorer segments of society who managed to mobilise sufficient financial capital to pay for their (initial) travel and care, using personal loans, donations from religious communities or fundraising by non-governmental organisations and diasporic networks.

The potential to earn substantial revenue is a key motivating factor for facilitation, and typically companies generate this in the form of commissions – a predetermined proportion of hospital fees which are paid to a facilitation company after completion of treatment and settling of the medical bill. There were respondents in both settings who spoke of receiving 20–30% of the fees paid by a user, but in Delhi there were also reports of some providers offering much larger commissions (of up to 50% of fees) in an effort to attract the users that facilitation companies promise to bring. In both settings there were companies which reported using alternative forms of payment such as fixed-rate one-off fees which are paid by users or by providers, and one respondent in London described a user subscription model their company had adopted to provide ongoing high-end ‘concierge’ facilitation support for companies and wealthy individuals.

The testimonies of respondents in both settings point to an area of activity that has become “saturated by agents” seeking to earn income through facilitation work. Although companies in both settings had been active in healthcare facilitation for several years, with one company in London that had been working internationally for 20 years, many companies were more recent entrants to the sector – approximately one-third (7/22) of the facilitation companies interviewed during the research had been founded since 2015. Companies had been founded by people with backgrounds in medicine, or in hospital management and marketing, which is unsurprising given the commercial advantages of possessing appropriate knowledge and networks; others were established by people with little professional experience of healthcare but who had been trained in business management and had some personal knowledge of local healthcare systems. In one case, a respondent had begun healthcare facilitation work after identifying this as an opportunity to utilise a family member’s international connections and their own experience as a mortgage broker. Most of the companies sampled in the research (14/22) were small (with five or fewer employees) but some in Delhi had grown substantially since their founding and now occupied large office spaces with rows of employees and differentiated company roles, including positions for directors, business development managers, accounts managers, human resources officers, case workers and in some cases in-house medics and interpreters. Delhi is also the site of a large informal facilitation sector which competes in some areas with the formal sector, particularly for lower-income users, and to which I will return later in the article.

The emergence of healthcare facilitation markets in the two settings reflects the relative ease with which someone can embark on this work. A personal or professional contact in another setting was sufficient for some respondents to begin facilitation work, but expanding and maintaining flows of users involved deepening relations to ensure a stream of clinical referrals and/or past-user recommendations. Respondents described developing their referral networks through processes of building “trust” that began with meeting a new contact and then proceeding through attempts to filter out the potential fraudulent contacts and receive a small number of “test” users. While some of this relationship-building took place in person, for example through travelling or national and international events, social media has also proved useful: one international business manager in Delhi described how they had incorporated LinkedIn into their daily routine and aimed to add several people as new contacts each day in the hope of generating productive relationships. Some larger companies from Delhi have established offices in other countries to generate a stream of users, in one case employing a local doctor to legitimise the company’s work in the eyes of a resistant local medical community that was concerned with the outflow of users. There are risks involved in these multi-national expansion strategies however: one company founder described experiencing substantial personal financial loss after having to close in-country offices in two locations due to political instability and violence in those settings.

The range of services offered by some companies (beyond a core offering described above) is revealing of the competitive pressures they face and their attempts to market themselves as distinct within the sector. For some in Delhi this has meant hiring full-time doctors who verify the necessity of care being provided – a service seen as important in a wider context of mistrust in healthcare provision [45] – and others offered what they claimed to be a “fair and transparent” fee system that set them apart from less scrupulous rivals incentivised by commission payments. In London, one company taps into an ecological zeitgeist by offering an “eco-friendly” experience that offsets carbon emissions for users travelling internationally. Respondents in both settings employed terms like “concierge”, “boutique” and “personalised” to differentiate their services from other companies, and in London that extended to claims by two companies that they would accompany users on international trips and take them to dinner the night before an operation. Companies also distinguish themselves from others by focusing on particular specialisms, for example cosmetic surgery or organ transplants, which allows accumulation of knowledge and networks in this area. In some cases it also allows companies to focus on specialisms with the largest medical bills – what the founder of one such company described as the “high-ticket patients”.

#### Close connections and threats of obsolescence

Systems of brokerage rely on a premise that using a third party carries some advantage in comparison to more direct forms of exchange, even if this means incorporating the intermediary’s own need to generate revenue. If that relative advantage is no longer clear to the other parties, they may consider there to be value in bypassing an intermediary, as one company director in the study noted: “the hospital and the client will get rid of me in a blink [of an eye]”.

During the research, the relationship between providers and facilitation companies often appeared close in both settings, and one respondent from a hospital in Delhi even remarked that users often do not realise the workers from facilitation companies are not employed by their hospital. One route of entry to employment in Delhi’s larger facilitation companies involves previous work in local hospitals, although representatives from some facilitation companies in Delhi highlighted a deliberate hiring strategy to avoid recruiting former hospital workers because of concerns that close ties would damage user perceptions of their independence. There is a tension between the need to maintain close connections with providers *and* with users, and accusations of bias towards one group or the other risk undermining the intermediary position and the business it brings – a typical concern for participation in brokerage relations [46].

For their part, leading practitioners and hospitals have been keen to encourage close connections with facilitation companies. Promotional videos for one company in London featured leading surgeons, and one small company in Delhi had been specifically formed to help manage and extend user flows for one leading clinician. Company founders in London reported enjoying holidays in other countries hosted by provider partners, and glamourous celebrations and dinners with senior practitioners. Respondents in Delhi described similar celebratory activities, such as parties hosted by hospitals with free food and drink for companies and their workers at the time of festivals, including iftar parties during Ramadan – tacit acknowledgement of the value of India’s Muslim identities in supporting user flows from Middle-Eastern countries. One large hospital near Delhi offered on-site office space to its top ten facilitation companies, judged in terms of revenue generated; another had dedicated an entire floor to users supported by a specific facilitation company.

The close connections in Delhi represent a recent phase of collaboration that was reportedly preceded by a more hostile attitudes towards facilitation during the early 2010s. At that time, India’s leading hospital chains worked in collaboration with the Federation of Indian Chambers of Commerce and Industries (FICCI) to ban healthcare facilitation through a series of events one respondent labelled as “how to eliminate the tout”, invoking a derogatory term used to describe facilitation in India [11]. After pushback from facilitation companies, the eventual outcome was a grudging acceptance by hospital chains of the need for facilitation as a conduit for users and revenue, and efforts to ban facilitation were replaced with efforts to formalise it. FICCI launched its annual Medical Travel Value Awards which include a category for facilitation, and an accreditation system was introduced, under the auspices of the National Accreditation Board for Hospitals & Healthcare Providers (NABH). The NABH accreditation for facilitation requires companies seeking accreditation to provide information on issues such as tax registration, staff qualifications and professional experience, and the hospitals with which the company has signed memoranda of understanding. As of July 2019, 15 companies had received this accreditation including seven in and around Delhi [47], however a large number of facilitation companies were continuing to operate without this accreditation, suggesting a mixed attitude towards this attempt at formalisation.

Perhaps the greater threat to the intermediary position of facilitation companies in both contexts is the expansion of providers’ marketing apparatus which aims to increase direct user flows, in some cases reportedly offering discounts to users who access them directly rather than via an intermediary. The growth of inward medical travel to these settings has been accompanied by the development of dedicated hospital administrative systems that encourage and organise user flows. London’s large public and private hospitals have international marketing operations, as have Delhi’s private hospitals, some of which have representatives based in booths in the international airport’s arrivals area to meet incoming travellers. Hospital marketing teams are responsible for developing their own referral networks in other countries and pursue agreements with companies who can sponsor healthcare and travel for workers. In some cases they can tap into the referral networks of facilitation companies in order to bypass those companies, offering better renumeration, as one respondent noted, “hospitals can pay them [in-country agents] more than me”. One recent trend is for hospitals to pursue telemedicine and perform clinical activities in other countries as a route for directly accessing users without the need for user travel, although in some cases this transnational expansion offers new opportunities for facilitation companies too.

#### Pursuit of “virgin markets”

One response to the threat of obsolescence posed by provider marketing has been for facilitation companies to adapt and consolidate their intermediary positions, with one company founder in Delhi confidently stating “you can’t eliminate the middle-man”. This has meant finding new ways to emphasise the value of facilitation work to users and to providers: offering new services to users, pursuing growth and individuation of user referral networks – what one company director described as finding “virgin markets” for their work – and developing new forms of commercial mediation with providers.

Some facilitation companies have gone to significant lengths to enhance their offer to users in order to protect their intermediary position in healthcare provisioning. There are companies in Delhi that employ in-house doctors to monitor and question the appropriateness of care as this is felt to contain costs and reassure users of the appropriateness of treatment in ways that emphasise the independence and value of the facilitation company. Companies in both settings claim to offer exclusive below-market packages or negotiated final medical bills, with one company director in Delhi describing his particularly adversarial approach to hospitals in which he would shout at hospital staff, “stare them down” and threaten to take users elsewhere in order to secure reduced fees.

There are attempts by facilitation companies to develop and offer new services to users, with “second-opinion” services an increasingly common offering. In London these services aim to tap into domestic discontent with clinical decisions in an under-resourced public healthcare system, as well as global demand for London-based healthcare expertise which can be delivered transnationally via facilitated teleconsultations. In Delhi, companies offering second-opinion services seek to address a breakdown in trust that has accompanied the expansion of commercially motivated healthcare and concern with unnecessary testing and treatment. The founder of one facilitation company targeting a domestic market in India told me how they deliberately used retired government doctors for their second opinion service as they were more likely to be seen as “non-commercialised”.

Company directors and business development managers engage in extensive searching to expand their word-of-mouth referral networks geographically. This is often international in nature, as representatives from facilitation companies travel widely to develop referral arrangements with individuals in other countries, but also includes domestic networks: companies in Delhi maintained connections with foreign embassies in India, pointing to a related system of brokerage performed by diplomatic missions, and some had developed arrangements with doctors in other Indian states to facilitate travel to Delhi for specialised treatments. Although countries in the Middle-East, Central and South Asia and Africa have typically been the target for international activities, several respondents in both settings mentioned China as a perceived area for market growth: some were actively developing connections in the country, and one had recently launched a Chinese-language version of its website.

One of the criticisms levelled at the personal referral network model is that it becomes unwieldy and impossible to maintain the necessary personal relationships beyond a certain point, and some respondents saw their company’s future in business-to-business accounts (“B2B verticals”) with companies in other countries. Respondents highlighted referral arrangements they had developed with insurance and telecommunications companies in other countries by approaching the companies with proposals for how much money could be saved on employee healthcare by using the respondent’s company. One company in the UK appealed to its domestic business clients by citing the convenience of a “concierge” service for company employees who could call a “magic number” in case of any health concerns. Others had softer institutional agreements for referrals in which they appeared to be a preferred choice for travelling users, for example with non-governmental organisations who were sponsoring someone’s care on a charitable basis.

The growing number of facilitation companies who organise international trips for their partner healthcare providers is particularly revealing of attempts to embed intermediary positions in new areas of commercial activity. Respondents in London reported facilitating travel for dentists from central Europe to northern England and London, or surgeons from London hospitals to Russia and Central Asian countries; while those in Delhi described organising short-term intensive “camps” for medics from Delhi to treat users in African countries. These visits have multiple purposes: provision of basic consultations and treatments, in some cases on a commercial basis with revenue divided between the organisations involved; maintaining relationships between facilitation companies and their in-country representatives; and generation of referrals for more complex care in London or Delhi. Respondents claimed extensive roles in the organisation of these trips, including performing market analyses and approaching hospitals with detailed business plans for the trips based on identified treatment gaps, expected budget, and projections for how much money a hospital would make by boosting its presence in the target area. The directors of one facilitation company had seen such commercial promise in these activities that they purchased ophthalmology equipment for use in camps, avoiding the need to lease equipment from a healthcare provider.

For commercial healthcare facilitation in the study settings, the combined challenge of competition and obsolescence has fostered a pressured environment that demands continuous exertion to consolidate and extend intermediary activities. This is unsurprising when understood as an area of work with relatively few barriers to entry and which involves maintenance of brokerage relations for commercial exchange. In the next sections I focus on how this commercialised environment – what one respondent in London went as far as to describe as a “rat race” – is felt at a personal level for those working in this sector.

### Personal strains in facilitation

#### Labour and loss: “I feel used sometimes”

In the pressured environment described above, a burden of labour placed on the workers in facilitation companies to meet expectations for service delivery and expansion. Healthcare facilitation is a labour-intensive process involving repeated calls to users, their families and healthcare providers, to organise healthcare visits, travel and follow-up. Respondents in smaller companies placed emphasis on the importance of personally conducting extensive consultations with users in advance of care, to ascertain the user’s needs and preferences, and then to present and discuss available options. In the case of cross-border travel, many respondents visited users during their stay in London or Delhi and sometimes accompanied them for appointments. Case workers in Delhi reported an expectation that they meet users at the airport and visit them each day; a task that becomes increasingly difficult as their caseload increases, and if users are divided amongst multiple hospitals in the city.

For workers in Delhi this has translated into long working hours with detrimental physical and mental consequences. When I asked about how many days they worked per week, one case worker, employed on a rolling contract for a company in Delhi, explained that “there are no days I’m not working [ …] if we have to work we have to work”. They noted regularly having as little as 3–4 h’ sleep as a result of long working days and regular night-time pick-ups at the airport: “we generally don’t get sleep”. Another recalled a particularly busy period the previous year when they had been responsible for attending to 12 visitors in Delhi and so had spent each day continuously travelling between one user after another; they noted the difficulty of performing this work for anyone with caring commitments. Yet this takes place in a wider context of under- and un-employment, and as the first worker mentioned above went on to note, “[my family] are happy that I’m working”.

Respondents from London’s smaller companies made similar comments about the demands and burdens of facilitation work. One described users “shopping around” between facilitation companies and providers in ways that they felt exploited their goodwill; another explained how “upsetting” it was to devote time to talking through a prospective user’s motivations, aims and possible treatment options, only to then be bypassed once the user had sufficient information to book and travel directly: “I feel used sometimes”. Other respondents highlighted the burden of always needing to be available by phone to current and prospective clients, with one describing how the advent of WhatsApp and Skype meant they felt they had to be constantly available to their clients: “these days the pressure is immense.”

An additional burden of labour is created by attempts to reduce the risk of under-payment for facilitation companies, and many respondents reported instances of users and providers withholding some or all of their payments. For the smallest companies, run by one or two people, these non-payments incurred further labour and detracted from other areas of work as they had to “chase and chase” people to try to get money owed. The commission-based payment system used by many facilitation companies aggravates the vulnerability of their situation as this system enables the arbitrary withholding of monies by providers. In one extreme case reported by the founder of a small company in London, a provider in another country had withheld commission payments due for more than 20 users, resulting in significant short-term personal financial difficulties and leaving the company with little financial capital to perform further facilitation work. Unbeknownst to the respondent, the provider had been heavily indebted and needed the user fees to repay lenders, leaving nothing for the respondent’s commission: “it was him or me”.

In order to reduce the risk of such problems, many of the respondents running smaller companies reported employing various tactics to ensure users settled their bills in full. Some respondents directly or indirectly checked the ability of clients to pay, for example using information from visa applications to assess whether users could afford an increase of 10–15% on the expected hospital bills; others took an advance deposit payment which would be large enough to cover the facilitation company’s fees in the event of later problems with payment. One respondent had employed this second strategy particularly effectively, describing an example where they used a personal contact in a national airline to hold a user at a boarding gate until they had settled their bill. Mediating the payment of medical bills completely can ensure that the facilitation company retains control over finances, however this risks leaving the facilitation company out of pocket when a user is slow in settling their bill and a provider unwilling to wait for payment, and has legal ramifications if facilitators become liable for provider malpractice.

The larger facilitation companies based in Delhi use their case workers to ensure timely payments, placing added pressure on those workers. Respondents reported an expectation that case workers are in attendance during the billings process in hospitals, “to ease things” – in other words to ensure complete payment. In cases where the final medical bill was higher than initially quoted, and users reluctant to pay the additional fee, workers described expectations from hospital staff that they explain and justify to users the increased bill, as one noted: the hospital “will pressurise us” to ensure the full bill is paid.

#### Interpreters as interlopers: “we hire you to help us!”

The need for language interpretation services for many users of healthcare services in the study settings introduces an additional area of waged labour into the facilitation process and creates new points of tension. Although respondents in London noted working with interpreters on an ad hoc basis through professional agencies, it was those interviewed in Delhi who provided more detailed insights into the personal pressures for this work.

Interpretation services, in healthcare and in other sectors, are a well-known opportunity for wage labour amongst younger workers with foreign language skills in Delhi, and the wages and tips offered to interpreters can be a glamorous source of income for young people in Delhi’s educated middle-class. One business development manager recalled being inspired to join the “shiny” facilitation sector when at university after seeing a friend dressed in a suit and travelling in “a fancy car” for interpretation work. Another respondent knew of several Arabic studies classmates from their university in Delhi who had worked as freelance interpreters in the healthcare industry. They cited a well-trodden path in which these students become part-time interpreters, develop professional networks and better wages, and over time lose interest in their studies and become full-fledged self-employed facilitators; indeed the founder of one of the larger companies included in the study had started out as an interpreter.

There is a significant degree of responsibility afforded to interpreters by facilitation companies. The interpreter’s daytime vigil over the user is a safeguard against possible exploitation and ‘snatching’ practices (discussed in the next section), or as one respondent put it, interpreters can make sure “nobody will interact with them [the user]”. The value of this role for interpreters is evidenced by the lengths that facilitation company management goes to in order to maintain good relations with certain interpreters. One company director reported giving monthly payments to interpreters even when they had not used their services, to ensure that they behaved favourably towards that company in future – “they should be loyal to us”. Others had introduced salaried roles as in-house interpreters for languages like Arabic that bring a steady stream of users to Delhi, and one company had started to source some of its case workers in countries with less commonly spoken languages, such as Mongolian, whom the company would sponsor to live in Delhi and work in the company. While providing some enhanced employment security to workers, arrangements that include travel and accommodation in India also offer significant leverage to employers in their labour relations if these employment benefits can be later withdrawn.

Other accounts of work as an interpreter paint a less glamourous picture. Respondents who had worked as interpreters described being on-demand throughout the week, facing the challenge of understanding complex medical terms and unfamiliar dialects, and having to pass on poor prognoses and news of failed treatments. They cited instances of feeling blamed by users when treatment failed or when final medical bills were larger than expected, and of feeling like a “mediator” during disputes between users, facilitation companies and providers. There were reports of instances where freelance interpreters were employed on low wages as they were recruited via an intermediary contact who would then keep part of their wages. One interpreter reported the routine withholding of money from interpreters on the pretext that they were “not working well” but that in reality this was a mechanism for facilitation companies to underpay interpreters and increase their own revenue.

The relationship between facilitation company and interpreter was described as fraught. One respondent, a student from Eastern Africa studying in Delhi, explained that they had been motivated to work as an interpreter to help people from their home region to access advanced healthcare and had sympathy towards users whom they felt were being exploited by facilitation companies and hospitals, and this led to repeated conflicts with their employer. In one case they had supported a user to find a guesthouse that was half the price of one allocated by the facilitation company, only for the company’s case worker to call a contact in the user’s home country, who in turn called the user and told them to return to the original guesthouse and not to trust the interpreter. In another instance, the interpreter was docked 1 day’s wages for arguing with a case worker when a user could not afford to pay their final bill and the hospital threatened to call the police; they recounted how the worker had exclaimed that the role of the interpreter was to get the user to pay, not to help the user; “we hire you to help us!”

This tension also played out in interviews with respondents in facilitation companies. They told me of instances where interpreters falsely inflated users’ guesthouse bills to receive a commission from the guesthouse, and where interpreters had asked doctors to perform unnecessary tests in order to incur further charges for which the interpreter could receive a commission. During several interviews the term ‘interpreter’ was used to refer to people working in the more informal spaces of Delhi’s facilitation sector, and this fed into a wider environment of mistrust and accusations of “snatching” users discussed in the next section.

#### Collaboration, mistrust and “snatching”

There were some respondents who reported positive engagement and collaboration between facilitation companies, for example through sectoral, business and healthcare industry events. In London, respondents noted having informal arrangements to work with other facilitation companies in specific circumstances, such as when they needed someone with additional linguistic expertise. In Delhi, a more formal mode of collaboration has emerged – several facilitation companies have come together to form an “aggregator” company. This provides an umbrella branding and enhanced bargaining power with hospitals on the basis of carrying a larger volume of provider revenue as compared to the smaller individual units. One respondent from an aggregated company stated that this had enabled the constituent companies in their aggregator to negotiate preferential commissions with hospitals.

Yet the competitive pressures of facilitation have engendered much anxiety and mistrust within the sector. In London, one respondent described how they checked the website of rival companies regularly and felt pressure to reduce their own prices when they saw they were being undercut; another had considered “giving up” and quitting facilitation work because of the way users contacted multiple companies and played them off against each other. Respondents noted a distaste for “gimmicks” used by rival companies, such as claims of ‘free’ accommodation or flights, or money-back guarantees for in vitro fertilisation. There were also concerns with how people working in rival companies attempted to develop relationships with the same providers in order to take business from the respondent’s company: one noted how a company had tried to contact surgeons they work with in central Europe to develop similar commercial arrangements; another reported that a rival used a list of providers from the respondent’s website to develop their own commercial arrangements with the same providers.

Respondents in Delhi reported a different manifestation of these pressures: what one case worker called the “snatching” of users. This refers to instances where one company makes administrative arrangements for someone to travel to India for healthcare, only for the user to avoid this company’s representative at the airport and instead take up the services of a rival. In a winner-takes-all system for commissions, the second company stands to receive the entire commission for the user’s healthcare fees. Respondents described “disheartening” experiences and the efforts taken by workers to try to find a user by calling them, sponsors and other contacts in the country of origin, and even calling around hospitals in Delhi. Often this snatching was explained as something performed by young men from Iraq and central Asian countries – ‘interpreters’ who were living in Delhi as refugees or on student visas and who could draw on shared cultural and linguistic understanding to strike up conversation with travellers in the arrivals area of the airport. Others suggested that ‘snatching’ was premeditated by users who were tactical in acquiring a travel visa to enter India and then pursued opportunities for cheaper care.

Discussion of the practice of ‘snatching’ in Delhi was accompanied at times with criticism of the commercial motivations of healthcare providers and a systemic unwillingness to intervene and prevent practices like this. Yet there were indications that some organisations have attempted to combat these practices, and this appears as much about financial loss and reputational damage to the wider industry as it is about the subsequent quality of care received by “snatched” users. A representative from the international marketing department in one large hospital told me how the hospital had introduced a points system for deciding which individual or company would be paid the commission for a particular user, based on receiving evidence that workers were involved in steps such as: receiving the first enquiry, organising the visa, and meeting the user at the airport. The hospital intervened in other conflicts too: one case worker from an NABH-accredited company described an incident where a worker at the hospital had encouraged the case worker’s client to move to a different guesthouse with assistance from a different facilitator. After receiving the original case worker’s complaint the hospital stepped in and threatened to ban the rival unless the user returned to their original facilitation company and guesthouse. The example points to a system of governance in facilitation that privileges the interests of Delhi’s formalised medical travel sector and facilitation companies over alternatives.

## Discussion

The study aims to provide new insights in an area of growing commercial activity in healthcare: commercial facilitation of access. This is an area characterised by commercial sensitivities which pose a challenge for research and which limited the range of data that could be collected; there were many emails and calls that went unanswered, and informal systems of brokerage proved particularly difficult to access. Despite these limitations, the focused approach to sampling permitted valuable in-depth data collection and the findings are likely to have salience in settings where commercial facilitation of access to healthcare is an established or emerging phenomenon.

The findings point to a strained existence for facilitation that stems from competition with rival facilitation companies and the activities of users and providers who seek to bypass, and even ban, facilitation. This extends previous research on challenges faced in facilitation: there is volatility in cross-border user flows [20], in an area ‘subject to shifts in fashion, finance, flight paths and medical technology’ ( [3], p. 532); and tensions that arise from the divergent interests of actors, including antagonistic relationships between facilitators and referring doctors [17, 18], and between interpreters and other actors [10, 19].

I have drawn attention to the strategies adopted by facilitation companies in the study settings which see them respond to pressured commercial environments by seeking to maintain and increase demand for their services. Recent work has looked in some detail at the mobilising work performed by facilitation companies to increase user flows [11], and here I point to the structural pressures of intermediary work as context for these activities. The findings in this article indicate strategic positioning by companies in ways that consolidate their intermediary roles: the adoption of differentiated roles and services that appeal to specific user groups, and the continuous search for new populations and activities to mediate, domestically and internationally.

The commercial environment for healthcare facilitation is marked by labour-intensive processes for organising healthcare (and its payment) in domestic and global markets, and by employment conditions displaying varying degrees of precarity. In spite of some examples of collaboration, this environment appears to incentivise exploitation and a commodification of users whose associated commissions are highly prized and who become targets for “snatching”. There are reported instances of exploitative practices being performed by facilitation companies in a range of settings: Holliday et al. [15] open their article with a powerful account of unethical practice as a healthcare user in Tunisia is left with unexpectedly limited support after the surgeon left with a suitcase of money and the facilitator followed soon after; Kaspar and Reddy [19] describe interpreters requesting inflated bills in order to receive higher commission, and taking healthcare users elsewhere if a provider refuses to oblige them; while several commentators have voiced concerns with the selective representation of information on facilitator websites [8, 48–50].

One of the proposed responses to these kinds of exploitative practices has been to encourage greater regulation and professionalisation in the sector [17]. Findings reported here suggest the need for careful consideration of how such regulation relates to existing stratifications in a sector where providers and users possess significant agency, and where exploitative practices take place in the context of a pressured commercial environment. The introduction of NABH accreditation in India marks an attempt by a corporate segment of the healthcare industry to formalise facilitation work in this setting, and in doing so promotes the interests of the better-resourced facilitation companies. It is unclear what effects this has had on the users who rely upon more informal networks of mediation to access alternative, possibly cheaper, forms of healthcare.

The brokerage analysis used here provides a basis for deeper theorisation on commercial healthcare facilitation. Systems of brokerage are conceived as dynamic arrangements in which intermediaries must continually justify and assert their role to avoid obsolescence and the loss of control over exchange and the revenue it brings [22]. These systems are characterised by tension between serving the needs of different parties [51], concerns with perceptions [46] and rivals [52], and the need to adapt and evolve activities in the face of shifting institutional landscapes [37]. By analysing commercial healthcare facilitation in terms of its brokerage relations, we can better understand the activities performed by this group of actors and the implications for healthcare provisioning.

In the findings I have drawn attention to the strains of work as an intermediary in the brokered relations for commercial healthcare facilitation: physical and mental burdens for labour, exploitative employment practices, and a wider atmosphere of mistrust. This echoes research on the vulnerability of intermediary positions in brokerage relations [53–55], and on the challenges posed by commercial work in the healthcare sector, for example the pressures faced by medical representatives [39, 40], and by health workers in private employment [56, 57]. The findings fit within a wider context of precaritisation for work [58], as pressure to be competitive in global markets has incentivised governments and companies to pursue flexibility in employment relations and working time arrangements [59, 60], but also chime with concerns regarding the growth of flexible labour in the ‘gig’ economy [61]. The locally and globally competitive nature of commercial healthcare facilitation, and the need to cater to the demands of healthcare consumers and providers, appears to encourage a particularly pernicious set of employment relations that rely on overwork and labour flexibility.

The growing body of social science research on cross-border travel for healthcare is providing valuable insights into the social relations and circulations that characterise one form of commercial healthcare facilitation [3–5], however the findings from this study point to several new directions for research: the role of diplomatic and business networks in facilitating access to care across borders, the new activities being mediated by facilitation companies in other countries, and the extent to which new technologies such as telemedicine are affecting facilitation work. Further research is needed to better understand the work of interpreters in commercial healthcare facilitation, and their exposure to exploitative employment relations.

The role of facilitation companies in domestic healthcare-seeking remains poorly understood and findings reported here point to opportunities for further research in this area. There are the domestic operations of facilitation companies that work in both global and domestic markets, and the concierge companies that hold aspirations to expand from an elite user-base to middle-class groups in many settings. There is also a corpus of online marketplace platforms, including ZocDoc, WeDoctor and Practo, which aim to facilitate access to healthcare in domestic settings and which have attracted significant investment from global financial capital to fuel their intra- and inter-national growth. These emergent forms of commercial healthcare facilitation have yet to be studied in detail and it will be important to understand the implications of their expansion for healthcare systems.

## Conclusion

The expansion of domestic and global markets for healthcare provisioning has created opportunities for third parties to position themselves as intermediaries who can connect users and providers of healthcare on a commercial basis. In Delhi and London this commercial mediation of access to healthcare is characterised by a pressured environment of competition and threatened obsolescence. Facilitation companies are faced with competition from rivals, and with users and providers seeking to selectively engage with, or bypass, their services, and so attempt to protect and expand their intermediary roles by offering new services, user flows and commercial activities that can ensure their continued relevance.

For people working in facilitation companies in these settings, competition and threats of obsolescence manifest in an onerous working environment in which they must meet the demands of users, providers and employers. There is a physical and mental burden incurred by the labour-intensive processes for generating and performing facilitation work, and for ensuring timely and complete payments. The presence of interpreters creates an additional component of work and new points for exploitation and tension in the facilitation process. In spite of some areas of collaboration, mistrust and anxiety appear to be key features for a commercial activity which incentivises the strategic out-manoeuvring of rivals.

Brokerage analysis provides valuable insights into the strategies and strains for commercial healthcare facilitation’s systems of mediation, in which facilitation companies extract revenue through their positioning in the centre of exchange relations and seek to motivate users and providers to continue mediated forms of exchange. The article indicates new avenues for further examination, including on the contributions to facilitation of interpreters, diplomatic and business networks, and new technologies, and on the growth of new forms of mediation in domestic and overseas settings. Future research in these areas will deepen our understanding of these influential systems for accessing healthcare.

## Data Availability

According to the Wellcome Trust’s Policy on data, software and materials management and sharing, all data supporting this study will be made openly available via the UK Data Service at https://www.ukdataservice.ac.uk/.
